# Dataset on photonic crystal fiber based chemical sensor

**DOI:** 10.1016/j.dib.2017.03.048

**Published:** 2017-04-08

**Authors:** Kawsar Ahmed, Bikash Kumar Paul, Sawrab Chowdhury, Md. Shadidul Islam, Shuvo Sen, Md. Ibadul Islam, Sayed Asaduzzaman, Ali Newaz Bahar, Mohammad Badrul Alam Miah

**Affiliations:** a Department of Information and Communication Technology (ICT), Mawlana Bhashani Science and Technology University (MBSTU), Santosh, Tangail 1902, Bangladesh; b Group of Bio-photomatiχ, Bangladesh; c Department of Software Engineering (SWE), Daffodil International University, Sukrabad, Dhaka 1207, Bangladesh

**Keywords:** Sen, Sensitivity response, CL, Confinement loss, *A*_eff_, Effective area, NA, Numerical aperture, *V*, *V*-parameter, MSS, Marcuse spot size, BD, Beam divergence, Beam divergence, Confinement loss, Chemical sensor data set, Index guiding photonic crystal fiber, Marcuse spot size, Numerical aperture

## Abstract

This article represents the data set of micro porous core photonic crystal fiber based chemical sensor. The suggested structure is folded cladding porous shaped with circular air hole. Here is investigated four distinctive parameters including relative sensitivity, confinement loss, numerical aperture (NA), and effective area (*A*eff). The numerical outcomes are computed over the E+S+C+L+U communication band. The useable sensed chemicals are methanol, ethanol, propanol, butanol, and pentanol whose are lies in the alcohol series (Paul et al., 2017) [1]. Furthermore, *V*-parameter (*V*), Marcuse spot size (MSS), and beam divergence (BD) are also investigated rigorously. All examined results have been obtained using finite element method based simulation software COMSOL Multiphysics 4.2 versions with anisotropic circular perfectly matched layer (A-CPML). The proposed PCF shows the high NA from 0.35 to 0.36; the low CL from ~10^–11^ to ~10^−7^ dB/m; the high *A*_eff_ from 5.50 to 5.66 µm^2^; the MSS from 1.0 to 1.08 µm; the BD from 0.43 to 0.46 rad at the controlling wavelength *λ* = 1.55 µm for employing alcohol series respectively.

**Specification Table**

**Table:** 

Subject area	Optical fiber
More Specific subject area	Photonic crystal fiber, Chemical sensor, Sensitivity, Confinement loss
Type of data	Numerical analyzed data
How data was acquired	Full vectorial finite element method (FV-FEM) based tool COMSOL Multiphysics version 4.2 with circular PML.
Data Format	Raw data.
Data accessibility	Data is within this article.

**Value of the data**

• Investigated data can assist engineers and researchers who are working with PCF based sensor gas and chemical senor.
• Proposed PCF based chemical sensor experienced with superior performances than previous PCF based sensor.
• For circular type׳s air cavities in both the cladding and core region, it can be easily fabricated by vastly used sol-gel method.
• Dataset is desirable for the benchmark of different chemical sensing application using PCF based sensor.

## 1 Data

This article describes the implementation of the photonic crystal based sensor with circular cladding with circular core. Investigation has done both for circular and elliptical holes. Table 1 is illustrating the dataset for PML depth on fiber properties; Table 2 is describing the variation of ±1% to ±2 around the optimum structure; Table 3 is describing the behavior of the PCF for different individual index based alcohol all are enumerated at the controlling wavelength *λ*=1.55 µm and Table 4 shows the Sellmeier coefficient for silica as the PCF forming material.

## 2 Design, materials and methods

**Fig. 1:**
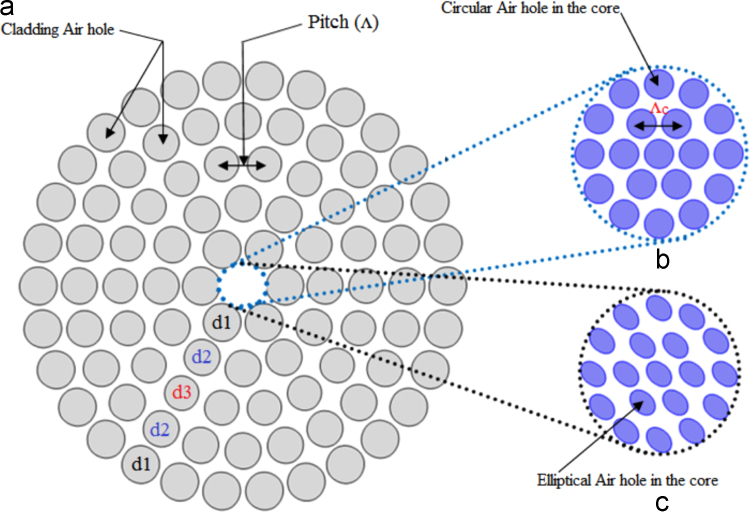
(a) Schematic end faced view of the proposed PCF (a) Cladding region, (b) Core with circular holes (c) Core with elliptical holes.

Fig. 1(a) is representing the schematic end face view of the proposed fiber [1]. The innermost part of the fiber is core, represented by Fig. 1(b) and (c) with circular air hole and elliptical air hole respectively. This region is holding two layers circular hole with same diameter. Nevertheless this region is also employed elliptical air holes for study the sensitivity performance and other modal parameters for the fiber. Outer region of the core of the fiber is cladding. The diameter of the hole is denoted by *d*_1_=*d*_5_=0.90∧_1_, *d*_2_=*d*_4_=0.86∧_1_, *d*_3_=0.82∧_1_.The fiber has containing finite number of air holes all playing as a role of dielectric medium in this outermost cladding region. The fiber creates symmetry for this fiber. Beside this and anisotropic circular perfectly matched layer is employed here to subdue unwanted incident electromagnetic ray acts as the boundary limit condition. The PML 10% PML depth is opted here [2].The hosting material for this PCF is pure silica is preferred here for its optical novelty [1], [2], [3], [4]. Fabrication is a vital issue for microstructure PCF. Different fabrication technique is used for their design flexibility reported in article [2], [3], [4].

**Fig. 2:**
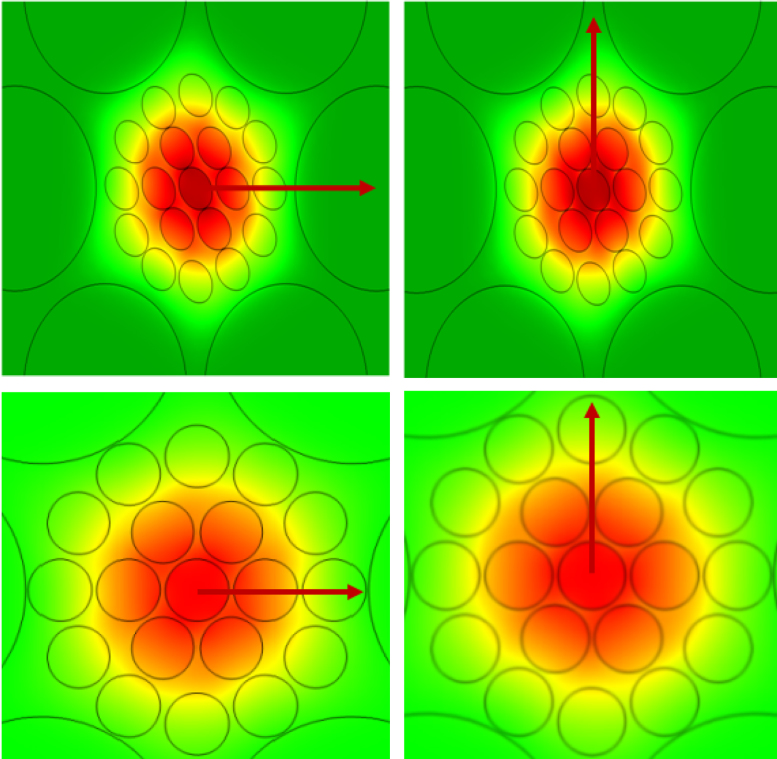
Light tightly confined inside the core for x and y-polarization at operating wavelength λ = 1.55 µm and n=1.354.

Fig. 2 demonstrates the modal intensity of the proposed PCF for both of elliptical and circular holes in core region in X-polarization and Y-polarization mode respectively. We have compiled two proposed PCF based sensor using FEM based commercial tool COMSOL Multiphysics version 4.2. Furthermore, finer mesh analysis is employed here to trace out the modal characteristics of PCF. Using this mesh analysis, it is found the number of vertex elements, boundary elements, total elements, and minimum element quality are 444, 3438, 30784, and 0.6773 respectively. For certain specific wavelength the light propagated within the core region. There aeries degree of freedom for operating wavelength. In controlling wavelength 1.55 µm is found 215665 degree of freedom. The background material of the PCF is silica. It has refractive index which is fully dependable on wavelength. For estimated different parameters for the proposed PCF Eqs. (1)–(10) are employed here, all are reported in the research article [1], [2], [3], [4], [5], [6], [7], [8], [9], [10], [11]. The relationship between refractive index and wavelength for silica is maintained by Sellmeier Eq. (1) as follows

(1) $$n_{\mathrm{silica}}\left(\lambda^{2}\right)=1+\sum_{j=1}^{k}\frac{B_{j}\times \lambda^{2}}{\lambda^{2}-C_{j}}$$

where, *λ* is the operating wavelength, *B*_j_ and *C*_j_ are the Sellmeier coefficient for silica noted in Table 4

The propagation constant β is generate here and abide by the following Eq. (2)

(2) $$\beta \;=\;n_{\mathrm{eff}}K_{0}$$

where, *K*_0_ = 2*π*/*λ*; *K*_0_ is the free space wave number. Due to the finite number of cladding air hole some light penetrate into the cladding region are liable for confinement or leakage loss. It can be enumerated from the imaginary part of the propagation constant β.

(3) $$L_{c}=8.686k_{0\;}I_{m}[n_{\mathrm{eff}}]\left[\frac{\mathrm{dB}}{m}\right]$$

where, *I*_m_ [*n*_eff_] is the imaginary part of the propagation constant. To realize the sensitivity response of the PCF it is necessary to compute the relative sensitivity coefficient r and it is maintaining the following Eq. (4)

(4) $$r=\frac{n_{r}}{R_{e}[n_{\mathrm{eff}}]}\times f$$

Here *R*_e_[*n*_eff_] is the real portion of *β*. But relative sensitivity coefficient *r* is closely involved with *f*. The *f* is the percent of energy that holds by the PCF cavities. There occurs energy conversation so the f can be expressed by Poynting׳s theorem and written as follows (5)

(5) $$f=\frac{\int_{\mathrm{tagetsample}}^{\;}R_{e}\left(E_{x}H_{y}-E_{y}H_{x}\right)\mathrm{dxdy}}{\int_{\mathrm{total}}^{\;}R_{e}\left(E_{x}H_{y}-E_{y}H_{x}\right)\mathrm{dxdy}}\times 100$$

In Eq. (5) numerator signifying the total power which is sense from target sample or target species and denominator representing total power of the PCF.

The effective area of the proposed can be formulated by the given Eq. (6)

(6) $$A_{\mathrm{eff}}=\frac{{\left(\iint {|E(x,y)|}^{2}\mathrm{dxdy}\right)}^{2}}{\iint {|E(x,y)|}^{4}\mathrm{dxdy}}$$

where, *E* is the transverse electric field vector of the fundamental mode and it is acquitted from proposed PCF. The source to fiber coupling efficiency is largely dependent on numerical aperture (NA). The NA of the PCF can be expressed as following Eq. (7) and NA is closely related with *A*_eff._

(7) $$\mathrm{NA}≅{\left[1+\;\frac{A_{\mathrm{eff}}}{\lambda^{2}}\times \pi \right]}^{{\;-}_{2}^{1}}$$

Certain of mode are propagating through the fiber. The figure of waveguide mode is ascertained by *V*-parameter or *V*_eff_. There also remains standard for *V*_eff_ which defines a fiber is multimode for *V*_eff_> 2.405 and otherwise it is single mode or mono mode. The *V*-parameter of the wave guide is calculated by Eq. (8)

(8) $$V_{\mathrm{eff}}\;=\;\frac{2\;\pi }{\lambda }\times \alpha_{\mathrm{eff}}\times \sqrt{n_{\mathrm{core}}^{2}-n_{\mathrm{cladding}}^{2}}$$

where, *α*_eff_ is the radius of the PCF core in µm unit. After determining the *V*_eff_ it is favorable to enumerate Marcuse spot size is expressed as the Eq. (9)

(9) $$W_{\mathrm{eff}}\;=\;R\times \left(0.65+\frac{1.619}{V^{3/2}}+\frac{2.879}{V^{6}}\right)$$

Beam divergence can be evaluated from Gaussian beam theory and it is denoted by *θ* in radian and calculated as follows Eq. (10)

(10) $$\theta \;=\;\tan^{\;-1}\left[\frac{\lambda }{\pi W_{\mathrm{eff}}}\right]$$

where, *θ* is in radian unit.

## Financial support

No Financial support was provided to any of the authors for this research work.

## Figures and Tables

**Table 1:** Variations on several PML depths to obverse the modal properties of the proposed PCF both of circular and elliptical holes at the operating wavelength *λ*=1.55 µm and *n*=1.354 (ethanol).

PML Depth	Sen (%)	CL [dB/m]	*A*_eff_ (µm^2^)	NA	*V*	MSS (µm)	BD (rad)	PCF Types
5%	65.18	7.57×10^−7^	5.61	0.36	4.92	1.06	0.44	Core with circular air holes
10%	65.18	9.45×10^−7^	5.61	0.36	4.92	1.06	0.44	
15%	65.18	1.23×10^−6^	5.61	0.36	4.92	1.06	0.44	
20%	65.18	1.44×10^−6^	5.61	0.36	4.92	1.06	0.44	
5%	57.11	6.65×10^−9^	5.59	0.35	4.62	1.02	0.45	Core with elliptical air holes
10%	57.11	8.20×10^−9^	5.59	0.35	4.62	1.02	0.45	
15%	57.11	7.80×10^−11^	5.59	0.35	4.62	1.02	0.45	
20%	57.11	4.10×10^−11^	5.59	0.35	4.62	1.02	0.45	

**Table 2:** Variations on overall parameter of proposed PCF around the optimum structure for circular and elliptical core respectively at the operating wavelength *λ*=1.55 µm, *n*=1.354 (ethanol); when core filled with aqueous analytes.

Variation	Sen (%)	CL [dB/m]	*A*_eff_ (µm^2^)	NA	*V*	MSS(µm)	BD (rad)	PCF Types
+2%	65.20	4.19×10^−^°^7^	5.68	0.34	5.02	1.08	0.43	Core with circular air holes
+1%	65.19	5.98×10^−07^	5.62	0.35	5.41	1.05	0.44	
Optimum	65.18	9.45×10^−07^	5.61	0.36	4.92	1.06	0.44	
-1%	65.17	6.25×10^−07^	5.66	0.35	4.87	1.05	0.44	
-2%	65.16	2.52×10^−07^	5.44	0.35	4.82	1.05	0.44	
+2%	58.51	9.44×10^−09^	5.56	0.35	4.72	1.03	0.45	Core with elliptical air holes
+1%	57.11	6.65×10^−09^	5.49	0.35	4.67	1.02	0.45	
Optimum	57.99	8.39×10^−09^	5.59	0.35	4.62	1.02	0.45	
-1%	57.09	8.09×10^−09^	5.39	0.35	4.58	1.01	0.45	
-2%	58.98	7.21×10^−09^	5.33	0.35	4.53	1.00	0.46	

**Table 3:** Modal characteristics analysis for alcohol series as core material, on the proposed PCF at the operating wavelength *λ*=1.55 µm for both type of PCF.

Variation	Sen (%)	CL [dB/m]	*A*_eff_ (µm^2^)	NA	*V*	MSS(µm)	BD (rad)	PCF Types
Methanol	64.46	6.57×10^−7^	5.65	0.35	4.72	1.07	0.43	Core with circular air holes
Ethanol	65.18	9.45×10^−7^	5.61	0.36	4.92	1.06	0.44	
Propanol	65.95	2.04×10^−10^	5.55	0.35	5.09	1.05	0.44	
Butanol	66.35	1.50×10^−10^	5.52	0.35	5.18	1.05	0.44	
Pentanol	66.73	4.41×10^−11^	5.49	0.35	5.27	1.04	0.44	
Methanol	55.68	6.65×10^−09^	5.62	0.35	4.44	1.01	0.45	Core with elliptical air holes
Ethanol	57.11	8.20×10^−09^	5.59	0.35	4.63	1.00	0.46	
Propanol	58.17	7.80×10^−10^	5.56	0.35	4.78	0.99	0.46	
Butanol	58.74	4.10×10^−11^	5.54	0.35	4.87	0.98	0.47	
Pentanol	59.29	5.88×10^−10^	5.51	0.35	4.96	0.98	0.47	

**Table 4:** Sellmeier coefficient for silica (SiO_2_) at *T*=25 °C.

**Parameters**	**Constant**	**Parameters**	**Constant**
B_1_	0.6961663	C_1_	4.67914826×10^−3^
B_2_	0.4079426	C_2_	1.35120631×10^−2^
B_3_	0.8974794	C_3_	9.79340025×10^1^

## References

[1] Paul B.K., Ahmed K., Asaduzzaman S., Islam M.S. (2017). Folded cladding porous shaped photonic crystal fiber with high sensitivity in optical sensing applications: design and analysis. Sens. Bio-Sens. Res..

[2] Ahmed K., Morshed M. (2016). Design and numerical analysis of microstructured-core octagonal photonic crystal fiber for sensing applications. Sens. Bio-Sens. Res..

[3] Ahmed K., Morshed M., Asaduzzaman S., Arif M.F.H. (2017). Optimization and enhancement of liquid analyte sensing performance based on square-cored octagonal photonic crystal fiber. Opt. – Inter. J. Light Electron. Opt..

[4] Asaduzzaman S., Ahmed K. (2016). Proposal of a gas sensor with high sensitivity, birefringence and nonlinearity for air pollution monitoring. Sens. Bio-Sens. Res..

[5] Paul D., Biswas R., Bhattacharyya N.S. (2015). Predicting different losses of photonic crystal fibers in material and hetero-core domain. Opt. Mater..

[6] Ahmed K., Islam M.S., Paul B.K. (2017). Design and numerical analysis: effect of core and cladding area on hybrid hexagonal microstructure optical fiber in environment pollution sensing applications. Karbala Int. J. Mod. Sci..

[7] B.K. Paul, M.S. Islam, S. Chowdhury, S. Asaduzzaman and K. Ahmed, Porous core Photonic Crystal Fiber based chemical sensor, In Electrical and Computer Engineering (ICECE), in: Proceedings of the 9th International Conference on. IEEE251-254. 〈http://dx.doi.org/10.1109/ICECE.2016.7853903〉, 2016.

[8] Chowdhury S., Sen S., Ahmed K., Paul B.K., Miah M.B.A., Asaduzzaman S., Islam M.S., Islam M.I. (2017). Porous shaped photonic crystal fiber with strong confinement field in sensing applications: design and analysis. Sens. Bio-Sens. Res..

[9] Asaduzzaman S., Ahmed K., Bhuiyan T., Farah T. (2016). Hybrid photonic crystal fiber in chemical sensing. SpringerPlus.

[10] S. Asaduzzaman, M. Arif, K. Ahmed, P. Dhar, Highly sensitive simple structure circular photonic crystal fiber based chemical sensor. InElectrical and Computer Engineering (WIECON-ECE), IEEE International WIE Conference on. IEEE151-154. http://dx.doi.org/10.1109/WIECON-ECE.2015.7443884, 2015.

[11] S. Asaduzzaman, K. Ahmed, M.F. Arif, M. Morshed, Proposal of a simple structure photonic crystal fiber for lower indexed chemical sensing. InComputer and Information Technology (ICCIT), in: Proceedings of the 18th International Conference on. IEEE127-131. 〈http://dx.doi.org/10.1109/ICCITechn.2015.7488055〉, 2015.

