# Supplementary material for: Dataset on photonic crystal fiber based chemical sensor
Source: Data Brief. 2017 Apr 8;12:227–33. doi: 10.1016/j.dib.2017.03.048 (PMC5397104; doi:10.1016/j.dib.2017.03.048)
Supplement: Supplementary file 1 — Supplementary material [file mmc1.docx]

To Whom It May Concern

On behalf of all the authors, Me Kawsar Ahmed would like to ask you to consider our manuscript entitled “**Dataset on** **Photonic crystal fiber based chemical sensor**” for publication in your journal as research article. In this article a PCF structure is proposed with associated dataset which can be helpful to design a chemical sensor for environment or air pollution monitoring. The proposed PCF not only shows higher sensitivity but also shows lower confinement loss and comparatively high numerical aperture compare to the existing structures. The article is providing very significant information for designing PCF based sensor in the modern optical perspective.

This manuscript has not been published yet and not even under consideration for publication elsewhere. All the authors have read the manuscript and approved this for submission as well as no competing interests.

Sincerely,

Kawsar Ahmed

Member of IEEE, OSA, SPIE

Assistant Professor

Department of Information and Communication Technology

Mawlana Bhashani Science and Technology University

Santosh, Tangail-1902, Bangladesh

Web: <http://ict.mbstu.ac.bd/faculty-details.php?id=17>

Cell: +88 01558514862

Email: k.ahmed.bd@ieee.org; kawsar.ict@mbstu.ac.bd; kawsarit08050@gmail.com
